# Widely Targeted Lipidomics and Microbiomics Perspectives Reveal the Mechanism of *Auricularia auricula* Polysaccharide’s Effect of Regulating Glucolipid Metabolism in High-Fat-Diet Mice

**DOI:** 10.3390/foods13172743

**Published:** 2024-08-29

**Authors:** Li Wu, Yibin Li, Shouhui Chen, Yanrong Yang, Baosha Tang, Minjie Weng, Hengsheng Shen, Junchen Chen, Pufu Lai

**Affiliations:** 1 Institute of Food Science and Technology, Fujian Academy of Agricultural Sciences, Fuzhou 350003, China; xxj1963@163.com (L.W.); lyb9951@163.com (Y.L.); cshh1307@163.com (S.C.); 18960973035@163.com (Y.Y.); tbsty@126.com (B.T.); 13959119561@163.com (M.W.); hsh87@hotmail.com (H.S.); junchenccc@163.com (J.C.); 2 National R & D Center of Edible Fungi Processing, Fuzhou 350003, China; 3 Key Laboratory of Subtropical Characteristic Fruits, Vegetables and Edible Fungi Processing (Co-Construction by Ministry and Province), Ministry of Agriculture and Rural Affairs, Fuzhou 350003, China; 4 Fujian Province Key Laboratory of Agricultural Products (Food) Processing Technology, Fuzhou 350003, China; 5 Fujian Characteristic Agricultural Products Processing Technology and Economic Integration Service Platform, Fuzhou 350003, China

**Keywords:** *Auricularia auricula*, polysaccharide, high-fat diet, hypolipidemia, widely targeted lipidomics, gut flora

## Abstract

The role of *Auricularia auricula* polysaccharide (AP) in the regulation of glycolipid metabolism was investigated using a high-fat-diet-induced hyperlipidemic mouse model. In a further step, its potential mechanism of action was investigated using microbiome analysis and widely targeted lipidomics. Compared to high-fat mice, dietary AP supplementation reduced body weight by 13.44%, liver index by 21.30%, epididymal fat index by 50.68%, fasting blood glucose (FBG) by 14.27%, serum total cholesterol (TC) by 20.30%, serum total triglycerides (TGs) by 23.81%, liver non-esterified fatty acid (NEFA) by 20.83%, liver TGs by 20.00%, and liver malondialdehyde (MDA) by 21.05%, and increased liver glutathione oxidase (GSH-PX) activity by 52.24%, total fecal bile acid (TBA) by 46.21%, and fecal TG by 27.16%, which significantly regulated glucose and lipid metabolism. Microbiome analysis showed that AP significantly downregulated the abundance of the Desulfobacterota phylum, as well as the genii *Desulfovibrio*, *Bilophila*, and *Oscillbacter* in the cecum of hyperlipidemic mice, which are positively correlated with high lipid indexes, while it upregulated the abundance of the families Eubacterium_coprostanoligenes_group and Ruminococcaceae, as well as the genii *Eubacterum_xylanophilum*_group, *Lachnospiraceae*_NK4A136_group, *Eubacterium_siraeum*_group, and *Parasutterella*, which were negatively correlated with high lipid indexes. In addition, AP promoted the formation of SCFAs by 119.38%. Widely targeted lipidomics analysis showed that AP intervention regulated 44 biomarkers in metabolic pathways such as sphingolipid metabolism and the AGE-RAGE signaling pathway in the hyperlipidemic mice (of which 15 metabolites such as unsaturated fatty acids, phosphatidylserine, and phosphatidylethanolamine were upregulated, and 29 metabolites such as phosphatidylcholine, ceramide, carnitine, and phosphatidylinositol were downregulated), thereby correcting glucose and lipid metabolism disorders.

## 1. Introduction

A substantial body of literature indicates that a prolonged, high-fat diet can precipitate aberrant glycolipid metabolism, leading to hyperlipidemia and, subsequently, the development of atherosclerosis [1]. In recent years, hyperlipidemia and atherosclerosis have emerged as two significant risk factors for cardiovascular and cerebrovascular diseases [2]. The annual incidence of cardiovascular and cerebrovascular diseases reached 3 million in China annually, accounting for 45% of total deaths. The incidence rate is rising year by year, and the population at risk is becoming younger [3]. Currently, synthetic statin drugs are frequently utilized in clinical practice to inhibit cholesterol synthesis and reduce cholesterol levels. However, these drugs may potentially impair liver and kidney function, leading to adverse reactions such as rhabdomyolysis [4]. The development of novel natural lipid-lowering functional ingredients, including polysaccharides, sterols, and dietary fiber, has become a prominent area of research [5].

*Auricularia auricula* (L. ex Hook) is an edible and medicinal mushroom that is cultivated on a global scale. It is the fourth most cultivated edible mushroom in the world in terms of production, as evidenced by data [6]. *A. auricula* is a rich source of protein, polysaccharides, vitamins, minerals (especially calcium, iron, and magnesium), and other nutrients. Additionally, it exhibits several pharmacological activities, including antioxidant, hypolipidemic, antiatherosclerotic, and intestinal microflora-regulating properties [7]. Of these, the polysaccharide derived from *A. auricula* (AP) is the most significant bioactive compound, comprising monosaccharides such as rhamnose, xylose, glucose, mannose, galactose, and arabinose [8]. Prior research has demonstrated that polysaccharides derived from *A. auricula* display notable efficacy in reducing serum malondialdehyde (MDA) levels, enhancing superoxide dismutase (SOD) activity, and augmenting total antioxidant capacity in the mouse model [9]. *A. auricula* and its polysaccharides have been shown to reduce total cholesterol (TC), total triglycerides (TGs), low-density lipoprotein cholesterol (LDL-C), and the atherosclerosis index (AI) in hyperlipidemic rats while simultaneously increasing high-density lipoprotein cholesterol (HDL-C) [10]. The beneficial regulatory effect of AP on blood lipids may be related to alterations in the structure and metabolites of the gut microbiota. The large-molecule polysaccharides of *A. auricula* are poorly digestible in the small intestine and typically enter the cecum and colon as prebiotics for gut bacteria, where they produce beneficial short-chain fatty acids (SCFAs). Zhang et al. observed that AP can better enrich SCFA-producing bacteria, such as *Paraprevotella*, *Flavonifractor*, and *Clostridium IV*, while downregulating the abundance of pro-inflammatory bacteria, such as *Prevotella* [10]. The impact of the AP compound food (comprising approximately one-third of the total mass) on lipid metabolism in hyperlipidemic rats fed a high-fat diet (HFD) was investigated through intestinal lipidomics. A total of 42 lipid biomarkers were identified, with the levels of lysophosphatidylethanolamine (LPE), phosphatidylglycerol (PG), phosphatidylinositol 4,5-bisphosphate (PIP2), and phytosphingosine (phSM) exhibiting a reduction [11].

Domestic and foreign research reports mainly focus on extraction technology [12], structural characterization [13,14], and lipid-lowering biological activities of AP [8,9,15]. The biological activity of macromolecular polysaccharides is closely related to gut microbiota and their metabolism [10]. However, the lipid-lowering mechanism of AP based on the gut microbiome and metabolomics is still unclear. The lipid-lowering effect of AP on high-fat-diet-induced hyperlipidemic mice was evaluated, and its lipid-lowering mechanism was revealed based on the gut microbiome and metabolomics, providing a scientific basis for the development and promotion of functional foods containing *A. auricula* polysaccharides.

## 2. Materials and Methods

### 2.1. Materials and Reagents

*A. auricula* was procured from supermarkets. The detection kits were purchased from Nanjing Detection Biological Engineering Company. The rest of the reagents were analytically pure.

Preparation of AP: A specified amount of *A. auricula* powder was weighed, and distilled water was added at a liquid-to-solid ratio of 110 mL g^−1^. The mixture was then subjected to ultrasonic treatment at 540 W for 20 min at room temperature. Following this, 0.25% pectinase was added, and the pH was adjusted to 5.6. The mixture was incubated in a water bath at 49 °C for 2 h. After extraction, the temperature was increased to 80 °C for 30 min to inactivate the enzyme, and the supernatant was then separated and concentrated [12]. The concentrated solution was precipitated with 3 times the volume of 95% ethanol, and then the sediment was redissolved in distilled water and freeze-dried. The total polysaccharide content of AP was approximately 85.02%, as determined by the phenol–sulphuric acid method. The heavy average molecular weight (Mw) of AP was determined by high-performance gel permeation chromatography with differential detection (GPC-RI), and the relative molecular masses were determined by multi-angle laser light scattering (MALLS). AP was composed of three polysaccharides with molecular weights of 1,960,000 Da (71.43%), 277,000 Da (14.08%), and 132,000 Da (14.49%). The polysaccharides were hydrolyzed entirely with trifluoroacetic acid, followed by derivatization with 1-phenyl-3-methyl-5-pyrazolone (PMP). Qualitative and quantitative analysis were performed on an HPLC equipped with a Thermo C18 column (4.6 mm × 250 mm, 5 µm). AP mainly comprised seven monosaccharides, including mannose 11.66%, rhamnose 3.72%, glucose 12.98%, galactose 1.45%, xylose 1.53%, arabinose 0.40%, and fucose 0.23%.

### 2.2. Instruments and Equipment

Electronic balance (Sartorius, Göttingen, Germany); high-speed frozen centrifuge (TGL-16GR, Shanghai Anting Scientific Instrument Factory, Shanghai, China); automatic biochemical analyzer (Toshiba TBA-40FR, Tokyo, Japan); paraffin microtome (LEICA RM 2126, Shanghai Leica Instruments Co., Ltd., Shanghai, China); digital video camera (Nikon ECLIPSE E200, Shanghai Henghao Instruments Co., Ltd., Shanghai, China).

### 2.3. Animal Experiment

Sixty 6-week-old pathogen-free (SPF grade) male Kunming mice, weighing (20 ± 2) g at the end of the adaptation period, purchased from Fujian Medical University (Animal Certificate No. SCXK (Beijing) 2019-0008). Maintenance feed: 52% corn meal, 23% soybean meal, l1% wheat bran, 9% flour, 2% calcium salt, and 3% mixed vitamins. High-fat feed: 1% cholesterol, 10% egg yolk powder, 10% lard, and 0.2% bile salt were added to 78.8% of the maintenance feed. All feeds were provided by Beijing Huafukang Biological Technology Co., Ltd. The 60 mice were randomly divided into 6 groups: the control group (NFD), model group (HFD), simvastatin group (Simv), low-dose-of-AP group (LAP), medium-dose-of-AP group (MAP), and high-dose-of-AP group (HAP). The NFD group was given maintenance feed, and the other groups were given high-fat feed; the Simv group was gavaged with 10 mg kg^−1^ simvastatin, and the LAP, MAP, and HAP groups were gavaged with 30, 150, and 300 mg kg^−1^ AP, respectively, along with the same volume of sterile distilled water on the NFD and HFD groups for 8 weeks. The body weights were weighed periodically (every Monday) to guide the adjustment of the gavage volume of mice. Blood and tissue samples were collected at the end of the experiment after a 12 h fast. All methods aimed to minimize the suffering of experimental animals were performed following the animal ethics guidelines and approved by the Committee of Animal Ethics (FJATCM-IAEC2019020).

### 2.4. Organ Index and Body Weight

Fasting body weight (BW) was weighed for 12 h. After blood collection, the liver and epididymal fat were removed separately, and the liver was weighed after saline drenching and filter paper aspiration, while the epididymal fat was weighed directly. The organ index (liver index, epididymal fat index) was computed according to the formula [organ index = fresh weight of organ (mg)/body weight (g)].

### 2.5. Biochemical Parameter Detection

At the end of the experiment, blood (1.5 mL for each mouse) was collected from mice by removing their eyeballs. Serum was collected by centrifugation of whole blood at 4 °C and 3000 rpm for 10 min. The serum biochemical parameters were determined with an automatic biochemical analyzer, including TC, TG, LDL-C, HDL-C, FBG, and non-esterified fatty acid (NEFA). One week before the end of the experiment, 5–8 fecal pellets were collected from each cage. The liver and fecal homogenate were prepared for the determination (including the TC, TG, total bile acids (TBAs), NEFA, glutathione peroxidase (GSH-PX), SOD, and MDA of the liver, and the TC, TG, and TBA of the feces). The kits were used according to the operating procedures.

### 2.6. Histological Examination

The histopathological analysis of liver and epididymal fat samples was performed using the hematoxylin–eosin (H&E) staining method. Then, tissue sections were photographed using an optical microscope (Nikon ECLIPSE E200, Tokyo, Japan) [16].

### 2.7. Analysis of SCFAs

The extraction of SCFAs from cecum contents was carried out according to the report [17]. 7890 GC-QqQ-MS (Agilent, California, CA, USA) was employed to detect fecal SCFA concentrations.

### 2.8. Amplicon Sequencing

Cecum contents were obtained by cutting the cecal segment with a sterile surgical knife, squeezing out the contents with forceps and placing in a 2 mL sterile cryopreservation tube, then rapidly freezing in liquid nitrogen and storing at −80 °C for testing. The 16S rDNA sequencing was conducted on the IonS5TMXL sequencing platform (Thermofisher) of Novogene Co., Ltd. (Shanghai, China). The taxonomy analysis, heatmap, LDA score, cladogram, and CCA plot were performed on the Novomagic cloud computing platform.

### 2.9. Wide-Target Lipidomics Analysis

Serum was analyzed using an LC-ESI-MS/MS system (UPLC, ExionLC AD, https://sciex.com.cn/, accessed on 24 June 2021; MS, QTRAPE System, https://sciex.com/, accessed on 24 June 2021) [18]. The reversed-phase (RP) analysis was performed using an Accucore^TM^ C30 (2.6 µm, 2.1 mm × 100 mm; Thermo, Waltham, MA, USA) at 45 °C. Widely targeted lipidomics analysis was assisted by Beijing Allwegene Technology Co., Ltd. (Beijing, China). Multivariate statistical analysis was carried out with MetaboAnalystR (V 1.0.1) software. PCA, PLS-DA, OPLS-DA, and S-loading plots were computed and visualized using SIMCA-14.1 software (UMETRICS, Umeå, Sweden). R (Complex Heatmap) (V 2.8.0) software was used to draw the heatmap. Finally, pathway analysis was executed with the MetaboAnalyst 5.0 online platform [19].

### 2.10. Statistical Analysis

The statistical data were analyzed with one-way ANOVA analysis in SPSS 25.0, and significance analysis was used for Duncan’s multiple comparisons.

## 3. Results and Discussion

### 3.1. Body Weight and Organ Index

**Figure 1:**
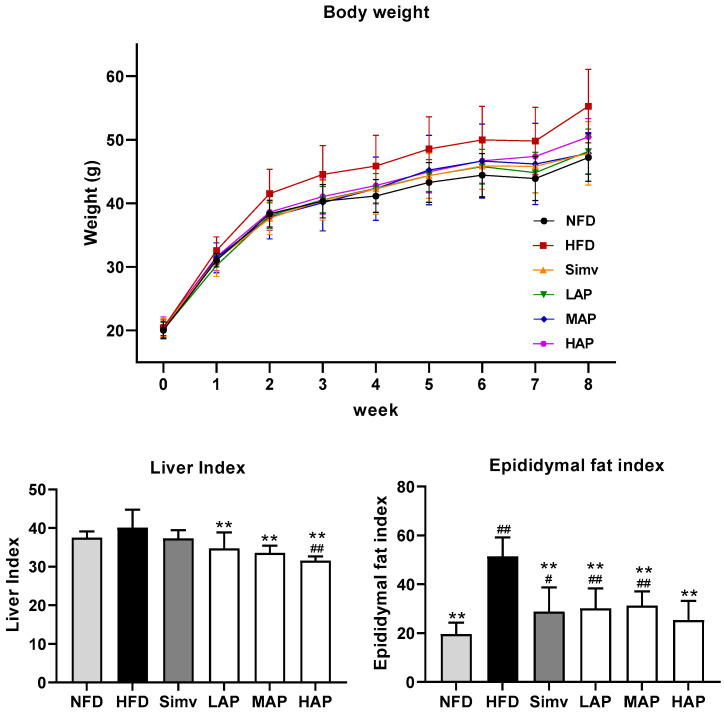
Body weight, liver index, and epididymal fat index. Data with different superscripts are significantly different at *p* < 0.05. ## *p* < 0.01 and # *p* < 0.05 versus the control group; ** *p* < 0.01 versus the model group.

As shown in Figure 1, the results of the BW dynamics of gavage for 8 weeks showed no difference in the initial BW of all groups, and the BW of the model group was significantly higher than that of the control group after 2 weeks of high-fat-diet feeding. The BW of the simvastatin group was close to that of the control group, and the BW of each AP treatment group was between that of the model and control groups. With the extension in gavage time, the effect of AP in controlling BW became more obvious. Compared with the BW of the model group, the BW of mice in the LAP and MAP groups was significantly reduced, and the control effect was close to that of the Simv group. The results showed that the liver index of mice fed with a high-fat diet had a tendency to increase, and compared with the liver index of the model group, the effect of AP showed a significant decrease in the liver index, with a decline of 13.40%. The epididymal fat index in the model group increased extremely significantly by 1.68 times compared with the control group. The epididymal fat index of AP was significantly reduced by 39.23% in the high-fat mice. The effect of reducing liver index and epididymal fat was close to that of the simvastatin group. The disorder of glucose and lipid metabolism caused by a high-fat diet is usually accompanied by significant obesity. Increased obesity promotes inflammation and oxidative stress, thereby promoting the development of hyperglycemia and hyperlipidemia [20]. AP significantly reduced the body weight of high-fat mice, significantly ameliorated the severity of epididymal and liver fat accumulation in mice, and had a certain anti-obesity effect. Similar results were found in a study using *A. auricula* powder to prevent obesity. Specifically, *A. auricula* powder replaced part of the diet to significantly reduce the body weight, liver index, and epididymal fat index in high-fat-diet-induced nutritional obesity model mice [21].

### 3.2. Serum Biochemical Parameters

**Figure 2:**
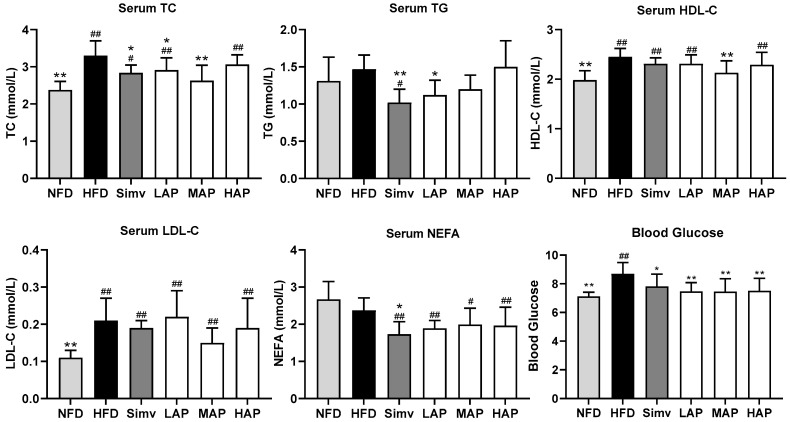
Serum biochemical parameters. Data with different superscripts are significantly different at *p* < 0.05. ## *p* < 0.01 and # *p* < 0.05 versus the control group; ** *p* < 0.01 and * *p* < 0.05 versus the model group.

Compared with the control group, serum TC and LDL-C were higher in the model group (*p* < 0.01), and TG tended to increase, indicating the model’s success. Compared with the model group, serum TC, TG, and NEFA were lower (*p* < 0.05) in the Simv group, TC and TG were lower in the LAP group (*p* < 0.05), and TC was significantly lower in the MAP group (*p* < 0.01). The results indicate that *AP* can reduce TC and TG. Compared with the fasting blood glucose (FBG) of the control group of 7.12 mmoL L^−1^, that of the model group of 8.69 mmoL L^−1^ was highly significantly increased after feeding the high-fat diet for 8 weeks. Compared with the model group, all AP treatment groups showed highly significantly reduced FBG (*p* < 0.01), and its effect was significantly better than that of the Simv group (Figure 2). Our findings align with those reported by Zhang [22], who developed an obesity model in male C57BL/6 mice using a high-fat diet. A subsequent intervention with 200 mg kg^−1^ of AP notably decreased both serum TG level and blood glucose concentration.

### 3.3. Liver Biochemical Parameters

**Figure 3:**
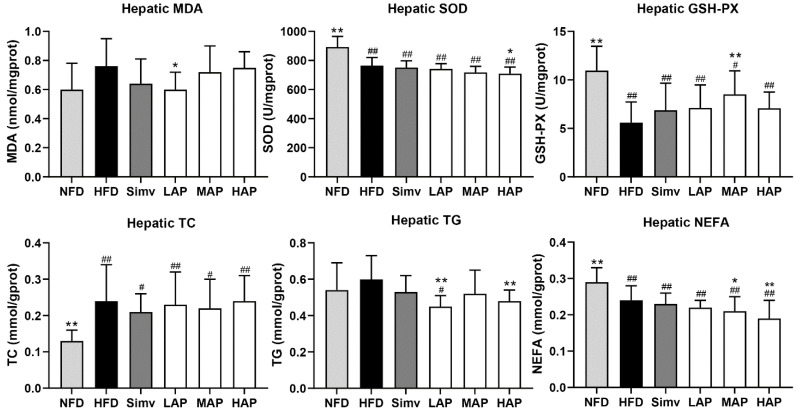
Liver biochemical parameters. Data with different superscripts are significantly different at *p* < 0.05. ## *p* < 0.01 and # *p* < 0.05 versus the control group; ** *p* < 0.01 and * *p* < 0.05 versus the model group.

In Figure 3, compared with the control group, the activity of hepatic SOD and GSH-PX was lower (*p* < 0.01), and the content of MDA only showed an increasing trend in the model group. Compared with the model group, the hepatic MDA was significantly lower in the LAP group, and the activity of hepatic GSH-PX increased dramatically in the MAP group. This indicates that AP had antioxidant activity that increased GSH-PX activity and decreased MDA content. The hepatic TC of the model mice was higher, while hepatic NEFA was lower than normal mice (*p* < 0.01). The hepatic TG of mice in the LAP and HAP groups was significantly lower, and the liver NEFA of mice in the MAP and HAP groups was considerably lower than that in the HFD group. The liver is an essential organ for lipid metabolism, including fat uptake, oxidation, synthesis, and secretion. Under normal circumstances, liver lipid metabolism maintains a dynamic balance. Excessive intake of lipids in a high-fat diet exceeds the liver’s ability to oxidize, synthesize, and transport, leading to liver fat hepatic steatosis and inflammatory infiltration [23]. In addition, literature reports suggest that oxidative stress plays a vital role in hyperlipidemia, and that the antioxidant activity of polysaccharides is closely related to their lipid-lowering activity [24]. The above liver biochemical indicators confirm that AP has hepatoprotective and antioxidant functions, which are beneficial for improving hyperlipidemia. The results are consistent with previous studies that report AP as being effective at simultaneously increasing GSH-PX activity and reducing MDA levels in mice with type 2 diabetes, thereby ameliorating oxidative stress-related damage [25].

### 3.4. Fecal Biochemical Parameters

**Figure 4:**
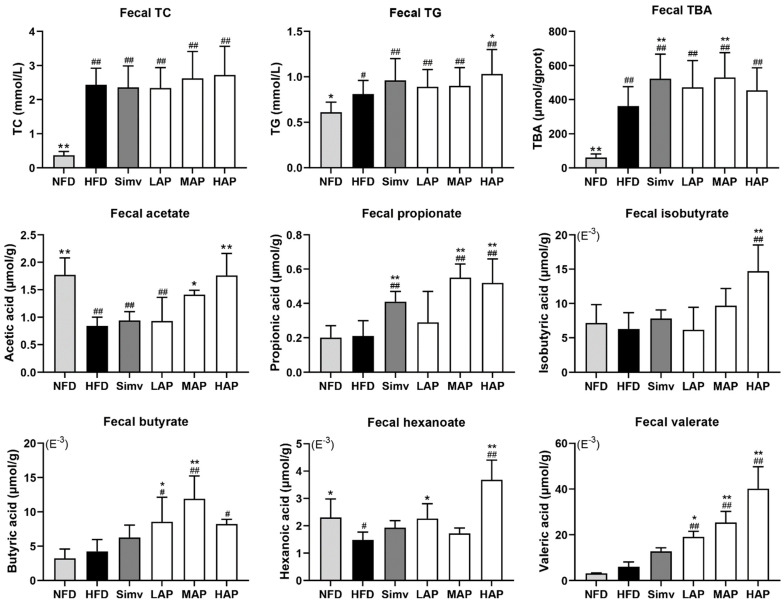
Fecal biochemical parameters. Data with different superscripts are significantly different at *p* < 0.05. ## *p* < 0.01 and # *p* < 0.05 versus the control group; ** *p* < 0.01 and * *p* < 0.05 versus the model group.

Compared to the NFD group, the model group exhibited a highly significant increase in fecal TBA, TC, and TG levels. Relative to the HFD group, fecal TBA levels were significantly higher in the Simv and MAP groups, while the fecal TG level was significantly higher in the HAP group. The result indicates the effect of AP in promoting the excretion of TG and TBA in feces. Compared with the control group, the content of acetate and hexanoate was significantly decreased in the model group, and the other four short-chain fatty acids did not change significantly. However, the acetate content increased in the MAP and HAP groups and the hexanoate content increased in the LAP and HAP groups (*p* < 0.05) compared to the model group. In addition, the valerate concentrations in the three AP intervention groups, propionate in the MAP and HAP groups, butyrate in the LAP and MAP groups, and isobutyrate in the HAP group were significantly increased compared with the model group (Figure 4). AP provides nutrients for SCFA-producing bacteria in the intestine, producing SCFAs such as acetic acid and butyric acid. The results for these SCFAs were similar to those reported in the literature and might be due to the regulation of gut flora by AP [26].

The domestic and international literature report that AP is an effective compound for lowering blood lipids [21,22,25,26]. On the one hand, AP may prevent cholesterol deposition and thrombosis in the blood and have a good auxiliary effect on preventing coronary atherosclerosis [8,11]. Its colloidal property has the physical impact of adsorbing fat from a high-fat diet and promoting fecal excretion of neutral cholesterol and bile acids [27]. Biochemical analyses from this experiment demonstrated that AP intervention modulated lipid metabolism by decreasing serum TC and TG, liver TG, and NEFA levels while increasing fecal SCFAs and promoting the excretion of TBA and TG in the feces.

### 3.5. The Morphology of Liver and Epididymal Adipocytes

**Figure 5:**
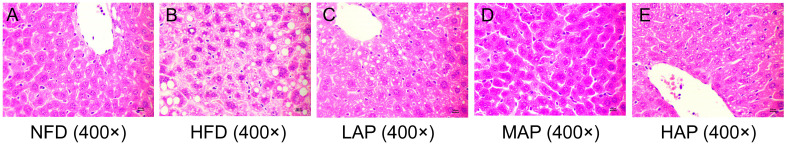
The histopathological changes in the hepatocyte. (**A**) NFD; (**B**) HFD; (**C**) LAP; (**D**) MAP; (**E**) HAP.

Using 400× HE stained sections (Figure 5), it was shown that liver cells in the NFD group were neatly arranged, with large and round nuclei in the center of the cells and clear cell borders. No lipid droplets were observed (lipid droplets were dissolved by ethanol and xylene during membrane formation, forming vacuoles) (Figure 5A). The liver cells in the HFD group were disordered, with circular vacuoles of varying sizes appearing in the cytoplasm. Some vacuoles were larger and squeezed the nucleus towards the edge, resulting in unclear cell boundaries and even fragmentation (Figure 5B). After LAP treatment, there was a slight amelioration in the morphology and structure of the liver cells (Figure 5C). The morphological improvement of liver cells was more significant in the MAP and HAP groups. The position of the cell nucleus was close to the center, and the cell border and vacuole area were significantly reduced (Figure 5D,E), which was consistent with the decrease in liver index (Figure 1).

**Figure 6:**
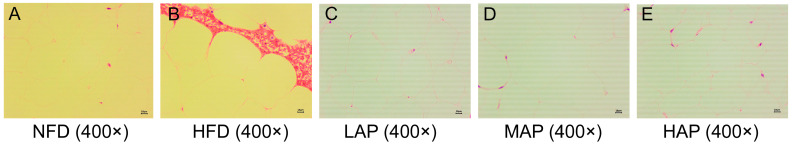
The histopathological changes in the epididymal fat. (**A**) NFD; (**B**) HFD; (**C**) LAP; (**D**) MAP; (**E**) HAP.

HE staining showed that the area of an epididymal adipocyte in the model group increased more significantly than in the control group (Figure 6). The number of adipocytes in the same area increased, and the area of adipocytes decreased as the dose of AP increased. Although there was no significant difference in body weight between mice on a high-fat diet and those on a maintenance diet, the liver and epididymal adipose indexes increased. A histological examination also revealed hepatocyte degeneration and an expansion in the epididymal adipocyte area. The AP-treated groups significantly reduced the liver and epididymal fat index, repaired hepatocyte damage degeneration, and reduced fat deposition in the liver and epididymis. Similar results were observed in a study examining the effects of *Auricularia auricula* and its polysaccharides on liver slices from rats fed a high-fat diet [10].

### 3.6. The Composition of Intestinal Microbiota

**Figure 7:**
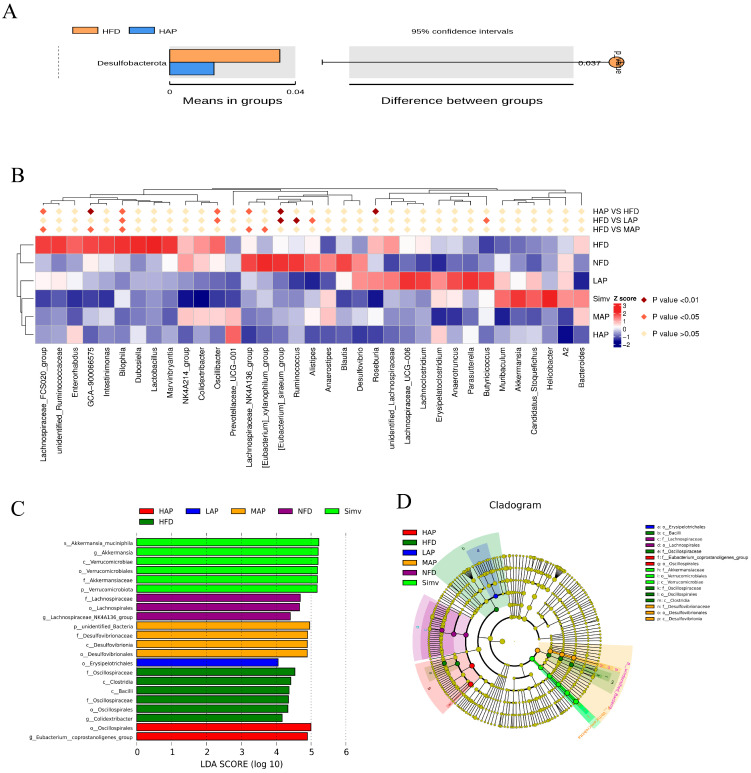
Differential bacteria of intestinal flora modulated by high-dose AP. (**A**) Differential bacteria in *t*-test model group and *A. auricula* polysaccharide high-dose group (*p* < 0.05, confidence interval 95%); (**B**) correlation analysis of all groups of samples with the top 35 ranked differential bacteria; (**C**) LDA score, (LDA > 4); (**D**) LDA tree.

In Figure 7A, the T-test shows that compared to the model group, the HAP group significantly downregulated the relative abundance of Desulfobacterota. Correlation analysis (Figure 7B) shows that compared with the NFD group, the HFD group significantly upregulated *Bilophila*, *Oscillbacter*, *Lachnospiraceae*_FCS020_group, *GCA-900066575*, etc., and considerably downregulated *Eubacterium_siraeum*_group, *Lachnospiraceae*_NK4A136_group, etc., which were reversed by AP (*p* < 0.05). Lefse analysis found that the bacteria with LDA > 4 in the NFD group were Lachnospiraceae, Lachnospirales, and *Lachnospiraceae*_NK4A136_group. The differential species in the HFD group were Oscillospiraceae, Clostridia, Bacilli, Oscillospirales, and *Colidextribacter* (LDA > 4). According to reports, the Desulfobacterota phylum and *Bilophila* genus are LPS-producing bacteria that can cause inflammatory reactions, metabolic disorders, and immune stimulation [28,29]. Supplementing the diet with the brown seaweed *Laminaria japonica*, rich in fiber, reduced the abundance of the obese bacteria *Oscillbacter* genus, leading to weight loss and prebiotic potential [30]. The *Eubacterium_siraeum*_group, *Lachnospiraceae*_NK4A136_group, and *Ruminococcus* are dominant microorganisms in zokors responsible for cellulose degradation [31]. The differential species in the Simv group were Verrucomicrobiota, Verrucomicrobiae, Verrucomicrobiales, Akkermansiaceae, *Akkermansia*, and *Akkermansia_muciniphila* (LDA > 4). According to reports [32,33], the relative abundance of Verrucomycota phylum in the high-fat-diet rats was significantly higher than that of normal mice. *Sarcodon aspratus* polysaccharide and orlistat intervention reduced or prevented high-fat-diet-induced obesity by upregulating the relative abundance of beneficial bacteria such as *Akkermensia*. The differential species with LDA scores greater than 4 in the high dose of AP treatment group were Oscillospirales and Eubacterium_coprostanoligenes_group. The Eubacterium_coprostanoligenes_group, one of the two critical bacteria genera in the fecal microecosystem of HFD mice, breaks down cholesterol into non-absorbable fecal sterols and excretes them with feces to reduce the cholesterol level of the body and play the role of lowering blood lipids [34,35]. Previous studies have shown that AP upregulated several low-abundance SCFA-producing bacteria, such as *Flavonifractor* and *Clostridium IV*, and downregulated pro-inflammatory bacteria *Prevotella* to counteract the effects of high-fat diet on gut microbes [10]. However, the experimental results differ from those of previous studies, which might be caused by the difference in the preparation process of polysaccharides, resulting in differences in structure and function.

**Figure 8:**
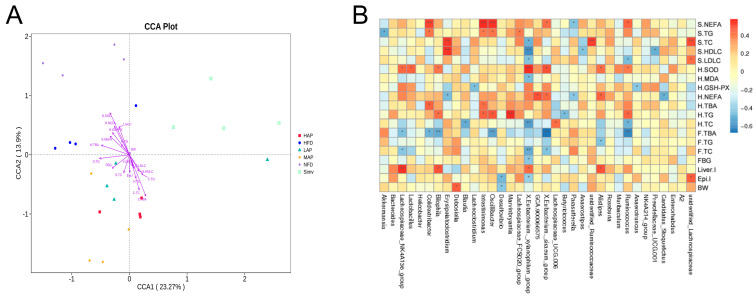
Environmental factor association analysis. (**A**) CCA analysis. (**B**) Heatmap of correlations between the top 35 gut microbiota taxa and environmental factors. Blue and red squares indicate negative and positive correlations, respectively. ** *p* < 0.01 and * *p* < 0.05 indicate that the difference was statistically significant.

The visible CCA analysis and heatmap show the relationship between 35 differential bacteria and environmental factors (Figure 8). *Eubacterium_xylanophilum*_group was positively correlated with hepatic SOD and negatively correlated with serum FBG, TC, LDL-C, hepatic MDA, and TC. Ruminococcaceae was positively correlated with hepatic SOD, while it was negatively correlated with serum LDL-C and hepatic TC. *Parasutterella* was negatively correlated with serum NEFA and hepatic NEFA. *Oscillobacter* was positively correlated with serum NEFA, TG, and hepatic TG, and negatively correlated with fecal TBA. *Bilophila* was positively correlated with hepatic TG, Liver I, and negatively correlated with hepatic SOD. *Desulfovibrio* was negatively correlated with Epi I and BW. Glucolipid metabolism and intestinal microecology were disturbed in high-fat mice, the abundance of *Eubacterium_xylanophilum*_group, Ruminococcaceae, and *Parasutterella* were downregulated, and the abundance of *Oscillobacter, Bilophila,* and *Desulfovibrio* were upregulated. After AP intervention, the abundance of those bacteria was reversed. A literature report showed that resveratrol significantly inhibited the relative abundance of *Bilophila* and *Desulfovibrio*, which are associated with diet-induced obesity [36]. Curcumin supplementation reduces the relative abundance of *Desulfovibrio*, a bacterium that produces endotoxins (also known as LPS) and alleviates high-fat-diet-induced hepatic steatosis and obesity [37]. Compared with the brothers and sisters of food-allergic children, the content of *Oscillobacter* in food-allergic children was enriched [38]. A high-meat, low-fiber diet increased the abundance of *Oscillobacter* compared with a high-fiber alternative to meat (mycoprotein) [39]. A fucoidan supplement upregulated the abundance of *Eubacterium_xylanophilum*_group and *Parasutterella* to improve ulcerative colitis [40]. A ganoderic acid-rich ethanol extract intervention significantly increased the relative abundance of *Eubacterium_xylanophilum*_group and Ruminococcaceae_UCG-009 to improve intestinal microbiota disorder in mice with alcoholic liver injury [41]. In addition, the *Eubacterium_xylanophilum*_group can break down complex polysaccharides and promote their absorption and utilization to produce beneficial short-chain fatty acids, particularly butyric acid [42].

### 3.7. Serum Widely Targeted Lipidomics

**Figure 9:**
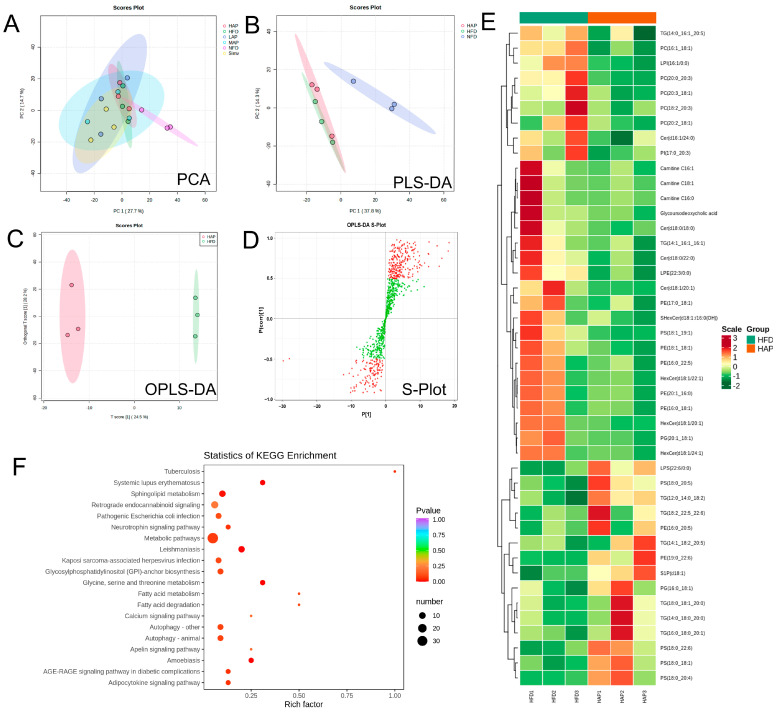
Serum widely targeted lipidomics analyzed with UPLC-ESI-MS/MS. (**A**) PCA; (**B**) PLS-DA; (**C**) OPLS-DA; (**D**) S-loading plot of OPLS-DA; (**E**) heatmap of significantly differential metabolites (VIP > 1.0, *p* < 0.05) between the model and HAP groups; (**F**) KEGG pathway analysis.

Figure 9A–C provide clear evidence that a high dose of AP can significantly alter the serum metabolic profile of mice fed a high-fat diet. Forty-four biomarkers were identified (*p* < 0.05, VIP > 1.0) (Figure 9D,E; Table S1), of which fifteen serum metabolites (including five TG, three PS, PE (16:0_20:5), etc.) exhibited a significant increase in concentration following AP administration. In contrast, 29 serum metabolites (including five PC, three Cer, three Carnitine, PI (17:0_20:3), etc.) demonstrated a significant decrease.

A reduction in phospholipid-containing PUFA, which is degraded through the lipid peroxidation process, may be a contributing factor to immune system disorders such as autoimmune diseases, systemic lupus erythematosus, and systemic sclerosis [43]. A similar phenomenon has been observed in hyperlipidemia, where polyunsaturated fatty acids and their corresponding phosphatidylserine are markedly diminished in rats fed a high-fat diet [44]. Ether phospholipids in sea urchins have been demonstrated to exert a regulatory effect on atherosclerosis in hamsters induced by high-fat diets, upregulating the levels of the lipid biomarkers phosphatidylethanolamine and phosphatidylinositol. AP has been shown to significantly upregulate the levels of unsaturated fatty acid phosphatidylserine and natural endogenous antioxidant phosphatidylethanolamine, thereby ameliorating hyperlipidemia in mice. Previously, it has been demonstrated that PC is the primary lipid species associated with disease activity and ursodeoxycholic acid response in patients with primary biliary cholangitis [45]. Additionally, there is evidence indicating a positive correlation between PC and subclinical atherosclerosis in individuals with type 1 diabetes [46]. Elevated levels of three distinct ceramide types were identified in the blood of Alzheimer’s disease patients [47]. Carnitine and related metabolites play a crucial role in mammals’ energy metabolism [48]. Non-targeted lipidomics has demonstrated that the reduction in ceramide, phosphatidylcholine, triacylglycerol, and phosphatidylinositol levels can facilitate the treatment of hypercholesterolemia and hyperlipidemia [49,50]. AP was observed to significantly downregulate the levels of phosphatidylcholine, ceramide, carnitine, and phosphatidylinositol, thereby improving hyperlipidemia in mice.

The metabolic pathway enrichment based on KEGG analysis showed that high doses of AP treatment mainly regulated glycerophospholipid metabolism, glycine, serine and threonine metabolism, fatty acid degradation, fatty acid metabolism, sphingolipid metabolism, the adipocytokine signaling pathway, and the AGE-RAGE signaling pathway (Figure 9F). Prior research has indicated a potential association between sphingolipid metabolism and age-related changes in muscle mass and functionality. This suggests that modulating the de novo biosynthesis of sphingolipids may represent a novel therapeutic avenue [51]. Previously, it was reported that 6′-O-caffeoyl butyric acid, extracted from *Vaccinium uliginosum*, may be an effective treatment for hyperlipidemia and thrombosis through the AGE-RAGE signaling pathway [52].

## 4. Conclusions

Based on physicochemical, microbiome, and widely targeted lipidomic analyses, the mechanism by which AP regulates glucose and lipid metabolism may involve chemical, biological, and physical aspects. Firstly, the chemical mechanism of AP regulates glycolipid metabolism through significantly reducing the liver index by 21.30%, epididymal fat index by 50.68%, FBG by 14.27%, serum TC by 20.30%, serum TG by 23.81%, liver NEFA by 20.83% liver TG by 20.00%, and MDA content by 21.05% in hyperlipidemic mice, while increasing the activity of liver GSH-PX by 52.24%. Secondly, the biological mechanism of AP regulates glucose and lipid metabolism through modulating the intestinal microbiota and increasing SCFA production by 119.38% (decreasing the abundance of the Desulfobacterota phylum, as well as the genii *Bilophila*, *Oscillbacter*, and *Desulfovibrio,* while improving the abundance of the families Eubacterium_coprostanoligenes_group and Ruminococcaceae, as well as the genii *Eubacterium_siraeum*_group, *Parasutterella*, *Eubacterium_xylanophilum*_group, and *Lachnospiraceae*_NK4A136_group). AP also addresses the disturbance of glucose and lipid metabolism by regulating biomarkers in metabolic pathways such as sphingolipid metabolism and the AGE-RAGE pathway, which includes the upregulation of unsaturated fatty acids, phosphatidylserine, and phosphatidylethanolamine and the downregulation of phosphatidylcholine, ceramide, carnitine, and phosphatidylinositol. Finally, the physical mechanism of AP regulates glucose and lipid metabolism based on the gel properties of AP, which can encapsulate digestive chyme, increase fecal TG by 27.16% and fecal TBA by 46.21%, and lastly, promote the excretion of lipid substances.

## Data Availability

The original contributions presented in the study are included in the article/Supplementary Material, further inquiries can be directed to the corresponding author.

## Supplementary Materials

The following supporting information can be downloaded at: https://www.mdpi.com/article/10.3390/foods13172743/s1. Names and classes of differential serum metabolites between HAP and HFD are in Table S1.
